# Comparative Analysis of Three Machine-Learning Techniques and Conventional Techniques for Predicting Sepsis-Induced Coagulopathy Progression

**DOI:** 10.3390/jcm9072113

**Published:** 2020-07-04

**Authors:** Daisuke Hasegawa, Kazuma Yamakawa, Kazuki Nishida, Naoki Okada, Shuhei Murao, Osamu Nishida

**Affiliations:** 1 Department of Anesthesiology and Critical Care Medicine, Fujita Health University School of Medicine, 1-98, Dengakugakubo, kutsukakecho, Toyoake, Aichi 470-1192, Japan; hasegawa.daisuke.0407@gmail.com (D.H.); nishida@fujita-hu.ac.jp (O.N.); 2 Department of Emergency Medicine, Osaka Medical College, 2-7 Daigakumachi, Takatsuki, Osaka 569-8686, Japan; wggdilp@gmail.com; 3 Department of Biostatistics Section, Center for Advanced Medicine and Clinical Research, Nagoya University Graduate School of Medicine, 65, Tsurumaicho, Showa, Nagoya, Aichi 466-8550, Japan; nishida@med.nagoya-u.ac.jp; 4 Department of Traumatology and Acute Critical Medicine, Osaka University Graduate School of Medicine, 2-2 Yamadaoka, Suita-shi, Osaka 565-0871, Japan; shumu20268271@gmail.com

**Keywords:** algorithms, artificial intelligence, disseminated intravascular coagulation, machine learning, sepsis

## Abstract

Sepsis-induced coagulopathy has poor prognosis; however, there is no established tool for predicting it. We aimed to create predictive models for coagulopathy progression using machine-learning techniques to evaluate predictive accuracies of machine-learning and conventional techniques. A post-hoc subgroup analysis was conducted based on the Japan Septic Disseminated Intravascular Coagulation retrospective study. We used the International Society on Thrombosis and Haemostasis disseminated intravascular coagulation (DIC) score to calculate the ΔDIC score as ((DIC score on Day 3) − (DIC score on Day 1)). The primary outcome was to determine whether the predictive accuracy of ΔDIC was more than 0. The secondary outcome was the actual predictive accuracy of ΔDIC (predicted ΔDIC−real ΔDIC). We used the machine-learning methods, such as random forests (RF), support vector machines (SVM), and neural networks (NN); their predictive accuracies were compared with those of conventional methods. In total, 1017 patients were included. Regarding DIC progression, predictive accuracy of the multiple linear regression, RF, SVM, and NN models was 63.7%, 67.0%, 64.4%, and 59.8%, respectively. The difference between predicted ΔDIC and real ΔDIC was 2.05, 1.54, 2.24, and 1.77 for the multiple linear regression, RF, SVM, and NN models, respectively. RF had the highest predictive accuracy.

## 1. Introduction

Sepsis is a life-threatening condition associated with the dysregulation of systemic host responses to microbial pathogens and leads to a disproportionate inflammatory response and multiorgan failure [1]. Sepsis-induced coagulopathy causes systemic failure and is associated with high mortality [2,3]. The main pathophysiological mechanisms of coagulopathy are inflammatory cytokine-initiated activation of tissue factor-dependent coagulation, insufficient control of anticoagulant pathways, and suppression of fibrinolysis. Together, these changes generate endothelial dysfunction and microvascular thrombosis, which can cause further organ dysfunction [4].

The diagnosis of sepsis-induced coagulopathy has gained increasing attention recently because of the increased risk of mortality associated with a greater severity of sepsis-induced coagulopathy [5]. Predictive modeling could facilitate the early identification of patients who are at high risk of progressive increase in the severity of coagulopathy. Moreover, predictive modeling could indicate the necessity of early administration of anticoagulant therapy. Currently, however, there is no established tool to predict the progression of coagulopathy in patients with sepsis.

Machine learning is an artificial intelligence technique that has shown to have higher accuracy in the detection and prediction of abnormalities in various clinical fields. Thus, we hypothesized that machine-learning techniques can contribute to a better predictive model for sepsis-induced coagulopathy [6,7]. The present study was conducted to create novel predictive models for the progression of coagulopathy in septic patients by using three machine-learning techniques. Furthermore, we evaluated the accuracy of the novel predictive models and compared it with that of conventional techniques to identify the most precise predictive model.

## 2. Methods

### 2.1. Study Design and Setting

This investigation was carried out as a post-hoc subgroup analysis of data recorded in a nationally representative dataset of critically ill patients that we obtained from a public, open data repository as published by Hayakawa et al. [7] in 2018 in accordance with the transparent reporting of a multivariable prediction model for the individual prognosis or diagnosis (TRIPOD) statement. The repository included data from a nationwide, multicenter, retrospective, cohort study, i.e., the Japan Septic Disseminated Intravascular Coagulation (JSEPTIC DIC) study [8], conducted at 42 intensive care units (ICUs) from 40 participating hospitals in Japan between January 2011 and December 2013. Patients were included in the registry if they were 18 years of age or older and diagnosed with severe sepsis or septic shock. In our subgroup analysis, we screened the cohort and included patients with adequate information on the diagnosis of sepsis-induced coagulopathy on days 1 and 3; we excluded patients with missing data for the main evaluation variables included in this study. This study was conducted in accordance with the principles of the Declaration of Helsinki, and the study protocol was approved by the Institutional Review Board of Osaka General Medical Center, Osaka, Japan (approval no. #25-2050).

### 2.2. Data Collection

In the original dataset, patients were followed up until hospital discharge or death. For this study, a dedicated case report form was developed, and the following information was obtained: age, sex, ICU admission route, pre-existing comorbidities, the primary source of infection, and therapeutic interventions against sepsis. We evaluated the severity of illnesses according to the Acute Physiology and Chronic Health Evaluation (APACHE) II score [9] and the Sequential Organ Failure Assessment (SOFA) score [10]. Seventy-three variables were collected in the public, open database; we excluded variables that were missing in more than 20% of the included patients and incorporated the remaining variables in the analysis dataset.

### 2.3. Definitions

According to the American College of Chest Physicians/Society of Critical Care Medicine consensus conference in 1991 [11], severe sepsis was defined as a suspected or proven infection, three or more signs of systemic inflammation, and more than one dysfunctional organ. In this study, we diagnosed sepsis-induced coagulopathy on the basis of the International Society on Thrombosis and Haemostasis (ISTH) overt disseminated intravascular coagulation (DIC) criteria [12]. The ISTH overt DIC criteria were adopted as proposed by the Scientific Subcommittee on DIC of the ISTH for platelet count, prothrombin time (PT), fibrin/fibrinogen-degradation products (FDP), and fibrinogen level. The fibrin-related marker was selected for the FDP values and scored by the cutoff levels and ranges previously published by Gando et al. [13] We calculated the ΔDIC score as ((DIC score on Day 3) − (DIC score on Day 1)). We defined “coagulopathy progression” as an increase in ΔDIC by one point or higher. The root mean square error (RMSE) in the ΔDIC score was defined as the difference between the predicted and the real ΔDIC.

### 2.4. Outcomes

The primary study outcome was whether the predictive accuracy of ΔDIC was more than 0. The secondary study outcome was the predictive accuracy of ΔDIC (the difference between the predicted ΔDIC and the real ΔDIC).

### 2.5. Statistical Analysis

To summarize baseline characteristics, continuous variables were expressed as median and interquartile range (25th–75th percentiles), and categorical variables were shown as number (percentage). To estimate the predictive performance, the original dataset was randomly divided into training dataset (70%) and test dataset (30%). The training dataset was used to develop each machine-learning algorithm, and the test dataset was used to evaluate the predictive accuracy. For the main analysis, we calculated the percentage of the correct prediction with regard to “coagulopathy progression” using conventional logistic regression and each of the machine-learning techniques, including the random forests (RF), support vector machines (SVM), and neural networks (NN).

For the secondary analysis, the RMSEs in the ΔDIC score were analyzed by multi-linear regression analysis, each machine-learning method was applied, and the RMSEs in each method were compared with each other. In the sensitivity analysis, the ability to predict sepsis-induced coagulopathy was verified using a dataset of all of the included septic patients with the ISTH overt DIC scores on days 1 and 3. This analysis was created by imputing the missing values with a mean value imputation using the same method.

In this study, when generating a multivariate logistic regression model and a multiple linear regression model, variable selection was undertaken by a stepwise method based on Akaike’s information criterion minimization [14]. This method was adopted as a baseline classical method for comparison with other machine-learning methods. RF is an ensemble learning algorithm that improves generalizability by integrating multiple weak learners on the basis of decision trees [15]. When used as a classifier, the importance of variables can be examined by using Gini coefficients. The larger the Gini coefficient, the more dispersed the result was; similarly, the smaller the Gini coefficient, the greater the divided result was. Therefore, it is possible to calculate how much the Gini coefficient has decreased by dividing specific variables. The SVM is a method for constructing two categories of pattern classifiers by using linear input elements [16]. The parameters of the linear input element are learned on the basis of identifying a margin-maximizing hyperplane that maximizes the distance to each data point. Even if a linear separation is impossible, the kernel function can be used to map a finite or an infinite dimensional feature space and conduct linear separation on that feature space. In this study, the radial basis function was applied for the kernel function. The NN is a mathematical model inspired by neural networks in the human brain [17]. The NN consists of an input layer, an output layer, and a hidden layer, with weights between these layers that indicate the strength of interneuron connections. Before constructing the neural network model, standardized scaling was applied to all of the covariates. In order to obtain a model with a small generalization error, it is necessary to properly adjust hyperparameters, such as the number of hidden layers, the number of nodes, and the optimizer. In this model, we performed 5-fold cross validation on the training data and optimized the hyperparameters of the number of hidden layers, the number of nodes, and the hyperparameters of the optimizer. We did a grid search on the following ranges and methods; one and two layers for the number of hidden layers, one to ten nodes for each layer, and the optimizer was chosen from limited memory BFGS, Stochastic Gradient Descent (SGD), and Adaptive moment estimation (Adam). Regarding the number of iterations, we did not use early termination. The maximum number of iterations was set to 10,000. For the activation function, we used the ReLu function. Neural network analysis was performed using Python 3.7.3, and other analyses were performed by using R (version 3.6.1; R Foundation for Statistical Computing, Vienna, Austria).

## 3. Results

### 3.1. Study Population and Included Covariates

**Figure 1:**
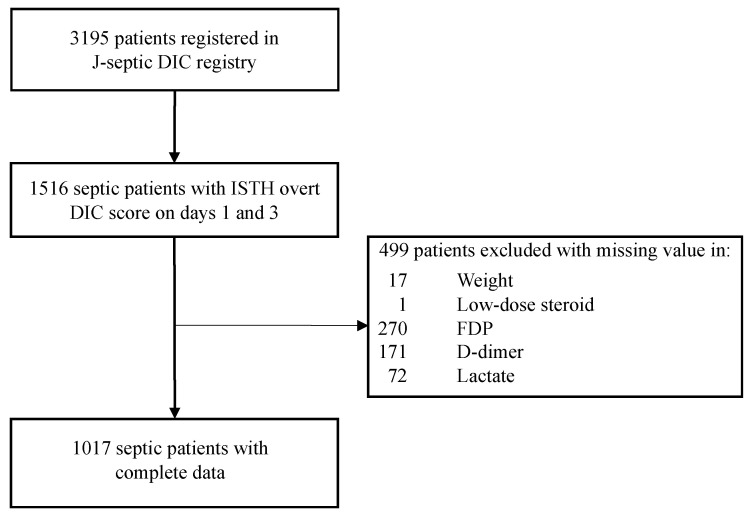
Schematic for patient screening, enrolment, and analysis. DIC, disseminated intravascular coagulation; ISTH overt DIC score, International Society on Thrombosis and Haemostasis overt Disseminate Intravascular Coagulation score; FDP, fibrin/fibrinogen-degradation product.

In the JSEPTIC DIC registry database comprising 3195 consecutive patients, a total of 1516 patients had an ISTH overt DIC score on days 1 and 3. After excluding patients with missing values for variables in the main analysis, we analyzed 1017 patients as the final study cohort (Figure 1). Of them, 601 (59.1%) were men, with a median age of 70 years (interquartile range (IQR): 60–79 years). The median APACHE II score was 23.0 (IQR; 17.0–29.0). The median ISTH overt DIC score was 4.0 (IQR: 3.0–5.0). The baseline characteristics of the study patients are shown in Table 1.

From the 73 variables we collected, antithrombin III was excluded because the number/rate of missing values was 442/29.2%, which was more than 20%; thus, 72 covariates were included in the final analysis in the three machine-learning techniques (Table S1). After the application of stepwise regression, covariates used in the linear and logistic regression analyses were decided. Tables S2 and S3 show the variables used in the main analysis from the multiple linear regression analysis and multiple logistic regression analysis, respectively; Tables S4 and S5 show the variables used in the sensitivity analysis from the multiple linear regression analysis and multiple logistic regression analysis, respectively.

#### 3.1.1. Prediction Accuracy with Conventional and Machine-Learning Approaches

The predictive accuracy rate, calculated as the percentage of the correct prediction rate of sepsis-induced coagulopathy progression on Day 3 using variables obtained on Day 1 from the multiple linear regression model, was 63.7%; however, the predictive accuracy rates of RF, SVM, and NN models were 67.0%, 64.4%, and 59.8%, respectively. As for the NN model, there was one hidden layer and six nodes. The chosen optimizer was SGD. Table 2 summarizes the contingency tables of the logistic regression analysis and three machine-learning methods.

**Figure 2:**
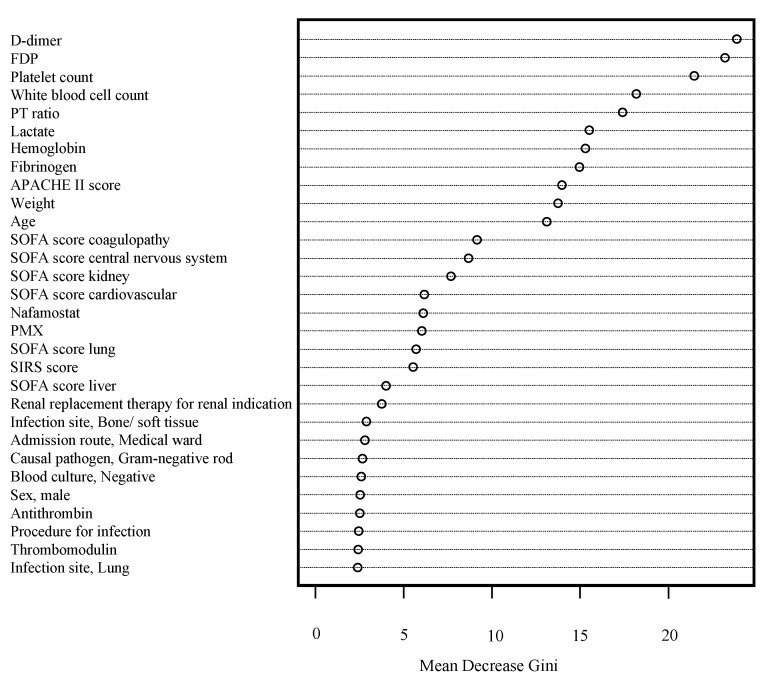
Importance of variables in a random forest plot examined by Gini coefficients. FDP, fibrin/fibrinogen-degradation product; PT, prothrombin time; APACHE, Acute Physiology and Chronic Health Evaluation; SOFA, Sequential Organ Failure Assessment; PMX, polymyxin B hemoperfusion; SIRS score, Systemic Inflammatory Response Syndrome.

**Figure 3:**
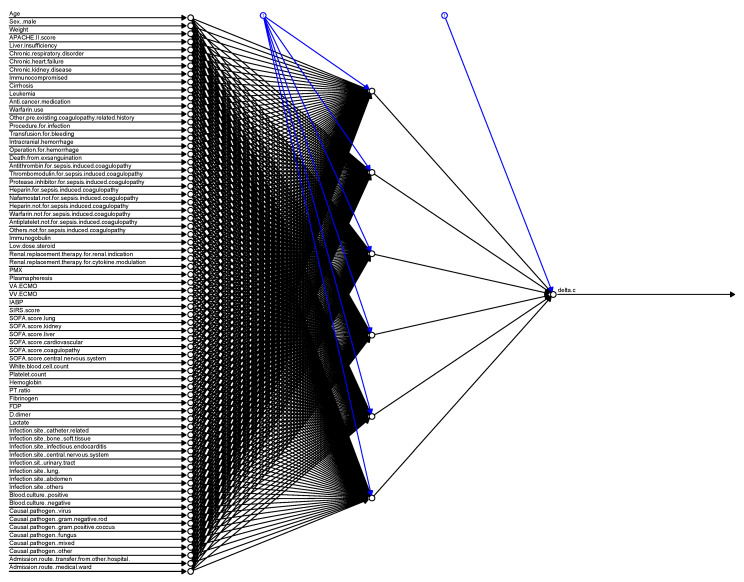
Construction of the neural network model for the prediction of sepsis-induced coagulopathy progression. APACHE, Acute Physiology and Chronic Health Evaluation; PMX, polymyxin B hemoperfusion; VA-ECMO, veno-arterial extracorporeal membranous oxygenation; VV-ECMO, veno-venous extracorporeal membranous oxygenation; IABP, intra-aortic balloon pumping; SIRS score, Systemic Inflammatory Response Syndrome; SOFA, Sequential Organ Failure Assessment; PT, prothrombin time; FDP, fibrin/fibrinogen-degradation product.

Figure 2 illustrates the strength of each variable to predict sepsis-induced coagulopathy progression in the RF model. D-Dimer, FDP, and platelet counts were the most attributable to the development of sepsis-induced coagulopathy. Figure 3 shows the construction of the NN in this analysis.

The RMSE in the ΔDIC score that was calculated as the difference between the predicted ΔDIC and the real ΔDIC using the multiple linear regression model was 2.05, whereas the RMSE in the ΔDIC scores using RF, SVM, and NN models was 1.54, 2.24, and 1.77, respectively. As for the NN model, the number of hidden layers was two, and the number of nodes was one in the first layer and nine in the second layer. The chosen optimizer was SGD. Among the methods, RF was the most accurate machine-learning technique.

#### 3.1.2. Sensitivity Analysis

After imputation involving 1516 patients, the predictive accuracy rates of coagulopathy progression using multiple linear regression, RF, SVM, and NN models were 66.2%, 72.1%, 68.4%, and 65.9%, respectively. As for the NN model, there was one hidden layer and one node. The chosen optimizer was Adam. Moreover, the RMSE in the ΔDIC scores using the multiple linear regression, RF, SVM, and NN models was 1.78, 1.47, 2.08, and 1.77, respectively. As for the NN model, the number of hidden layers was two, and the number of nodes was one in the first layer and five in the second layer. The chosen optimizer was limited memory BFGS. Similar to the main analysis, RF was the most accurate technique.

## 4. Discussion

To the best of our knowledge, this is the first reported study to explore the predictive accuracy of techniques evaluating the progression of sepsis-induced coagulopathy. With the use of machine-learning techniques and conventional multiple logistic regression analyses, coagulopathy progression on Day 3 was predicted with an accuracy rate ranging from 59.8% to 67.0% using variables that were obtained on Day 1. Similarly, with the use of machine-learning techniques and conventional multiple linear regression analyses, the difference between the predicted ΔDIC and the real ΔDIC on Day 3 ranged from 1.54 to 2.24 using variables obtained on Day 1. Among the tested techniques, RF was found to be the most accurate. However, the predictive accuracy was not sufficiently high for use in clinical practice.

Given the complex pathophysiology of sepsis-induced coagulopathy [18], the predictive accuracy could be increased by using machine-learning models that can incorporate the complicated relationships between covariates into consideration, in comparison with conventional techniques such as multiple linear/logistic regression models with simple linear functions [19]. Despite the slightly superior predictive accuracy of machine-learning techniques to that of conventional techniques, the predictive accuracy still requires improvements for it to be clinically applicable. We assumed that the sample size and the number of available covariates related to the development of coagulopathy were both inadequate to establish the required predictive accuracy. In particular, we could not collect data on the coagulation marker antithrombin III, which is an important marker for the pathogenesis of sepsis-induced coagulopathy [20]. Decreased antithrombin III activity is a consequence of excessive thrombin generation [21], increased vascular permeability [22], accelerated antithrombin III degradation [23], and impaired antithrombin synthesis [24]. However, due to the high rate of missing data, we could not include this marker in the post-hoc analysis. Furthermore, due to the lack of data, some of the important coagulation markers which directly affect the development of coagulopathy, such as thrombin-antithrombin complex, soluble fibrin, and prothrombin fragment1+2 [25] were not incorporated into the analysis. Thus, the limited availability of covariates related to coagulation markers might translate to a less elaborate model development to precisely predict the progression of coagulopathy in septic patients. Therefore, the establishment of a new model with more coagulation-related laboratory tests as covariates is necessary for better predictive ability.

We believe that the development of models to identify patients who are at high risk of developing coagulopathy in the early phase of sepsis is clinically important to predict the prognosis and is useful to select the best targets for the administration of agents that influence the coagulation cascade [5]. A recent randomized controlled trial failed to show the survival benefit of anticoagulation for sepsis-induced coagulopathy [26]. However, their result might be explained by the time window for the anticoagulant therapy [27] because anticoagulation might be most effective when administered in the early phases [28,29]. Thus, the prediction of coagulopathy development is clinically important for proper selection of patients and the time window for anticoagulation.

There are several limitations in this study. First, because this was a retrospective study, there remain the risk of residual confounding and the risk of type I error. Second, because the laboratory tests and their frequency of sampling differ among the participating study centers and are not standardized in Japanese hospitals, there were many missing values that are required for the diagnosis of overt DIC based on ISTH for the main analysis. However, we managed to include more than 1000 patients in the final analysis. Third, it is possible that variables such as vital signs including heart rate, blood pressure, and so forth, which were not used in this study, might have played a decisive role in the progression of sepsis-induced coagulopathy because of the complicated and heterogeneous nature of this disease entity. However, we used 73 variables, and we believe that this study presents the currently available best evidence for predicting the progression of sepsis-induced coagulopathy. Overall, prospective studies with a larger sample size and additional covariates are warranted to provide more definitive data to validate our findings.

## 5. Conclusions

The prediction of coagulopathy progression in patients with sepsis was explored with several machine-learning and conventional approaches. The methods studied have predictive accuracies ranging from 59.8% to 67.0%. Further research should increase the predictive accuracy of these machine-learning techniques to ensure they are fit for clinical application.

## Figures and Tables

**Table 1:** Baseline characteristics of all patients (*N* = 1017).

**Patient Characteristics**	
Age (years), mean (range)	70.0 (60.0–79.0)
Sex, male, *N*/%	601/59.1%
Weight (kg), mean (range)	56.0 (48.5–65.4)
**Pre-Existing Comorbidities**	
Liver insufficiency, *N*/%	49/4.82%
Chronic respiratory disorder, *N*/%	38/3.74%
Chronic heart failure, *N*/%	66/6.49%
Chronic kidney disease, *N*/%	73/7.18%
Immunocompromised, *N*/%	178/17.5%
**Infection Site**	
Catheter related, *N*/%	16/1.57%
Bone/soft tissue, *N*/%	158/15.5%
Infectious endocarditis, *N*/%	25/2.46%
Central nervous system, *N*/%	22/2.16%
Urinary tract, *N*/%	128/12.6%
Lung, *N*/%	230/22.6%
Abdomen, *N*/%	364/35.8%
Others, *N*/%	20/1.97%
**Illness Severity Score on Day 1, Mean (Range)**	
APACHE II score	23.0 (17.0–29.0)
SIRS score	3 (2–4)
SOFA score, lung	2 (1–3)
SOFA score, kidney	2 (0–3)
SOFA score, liver	0 (0–1)
SOFA score, cardiovascular	3 (1–4)
SOFA score, coagulopathy	1 (0–2)
SOFA score, central nervous system	1 (0–2)
ISTH overt DIC score	4.0 (3.0–5.0)
**Laboratory Data on Day 1, Mean (Range)**	
White blood cell count (×10^3^/μL)	11.8 (4.70–19.3)
Platelet count (×10^4^/μL)	111 (56.0–180)
Hemoglobin (g/dL)	10.5 (8.9–12.2)
PT ratio	1.36 (1.20–1.62)
Fibrinogen (mg/dL)	386 (263–544)
FDP (μg/mL)	20.0 (11.0–40.0)
D-dimer (μg/mL)	8.90 (4.30–20.80)
Lactate (mmol/L)	2.80 (1.80–5.04)

Data are presented as the median and interquartile ranges (25th–75th percentile) or as absolute frequencies with percentages. APACHE, Acute Physiology and Chronic Health Evaluation; SIRS score, Systemic Inflammatory Response Syndrome; SOFA, Sequential Organ Failure Assessment; ISTH overt DIC score, International Society on Thrombosis and Haemostasis overt Disseminated Intravascular Coagulation score; PT, prothrombin time; FDP, fibrin/fibrinogen-degradation product.

**Table 2:** Prognostic accuracy with each predictive model.

Statistical Analysis	TP	FP	FN	TN	Accuracy
**Logistic Regression**	44	33	78	151	63.7%
**SVM**	40	34	82	150	64.4%
**RF**	38	17	84	167	67.0%
**NN**	46	47	76	137	59.8%

TP, true positive; FP, false positive; FN, false negative; TN, true negative; SVM, support vector machine; RF, random forest; NN, neural network.

## Supplementary Materials

The following are available online at https://www.mdpi.com/2077-0383/9/7/2113/s1, Table S1: Covariates included in the three machine-learning techniques, Table S2: Variables included in the analysis, their estimated values, and *p*-value in multiple linear regression analysis with complete data, Table S3: Variables and their odds ratios in multiple logistic regression analysis with complete data, Table S4: Included variables, their estimated value, and *p*-value in multiple linear regression analysis with imputation, Table S5: Variables and their odds ratios included in multiple logistic regression analysis of imputation.
