# Supplementary material for: Comparative Analysis of Three Machine-Learning Techniques and Conventional Techniques for Predicting Sepsis-Induced Coagulopathy Progression
Source: J Clin Med. 2020 Jul 4;9(7):2113. doi: 10.3390/jcm9072113 (PMC7408668; doi:10.3390/jcm9072113)
Supplement: Supplementary file 1 [file jcm-09-02113-s001.zip › TableS1.pdf]

Table S1. Covariates included in the three machine-learning techniques PMX, polymyxin B hemoperfusion; VA-ECMO, veno-arterial extracorporeal membranous oxygenation; VV-ECMO, veno-venous extracorporeal membranous oxygenation; IABP, intra-aortic balloon pumping, APACHE, Acute Physiology and Chronic Health Evaluation; SIRS, Systemic Inflammatory Response Syndrome; SOFA, Sequential Organ Failure Assessment; PT ratio, prothrombin:time ratio; FDP, fibrin/fibrinogen-degradation product

Patient characteristics	Age, Sex, Weight
Pre-existing comorbidities	Liver insufficiency, Chronic respiratory disorder, Chronic heart failure, Chronic kidney disease, Immunocompromised
Pre-existing coagulopathy-related history	Cirrhosis, Leukemia, Anticancer medication, Warfarin use, Other
Infection site	Catheter related, Bone/soft tissue, Infectious endocarditis, Central nervous system, Urinary tract, Lung, Abdomen, Others
Blood culture	Positive, Negative, No attempt
Causal pathogen	Gram-negative rod, Gram-positive coccus, Fungus, Virus, Mixed, Other, Unknown
Admission route	Medical ward, Emergency department, Transfer from other hospital
Bleeding event	Transfusion for bleeding, Death from exsanguination, Intracranial hemorrhage
Anticoagulant therapy for	Antithrombin, Thrombomodulin, Protease inhibitor, heparin

sepsis-induced coagulopathy	
Anticoagulant therapy not for sepsis-induced coagulopathy	Nafamostat, Heparin, Warfarin, Antiplatelet, Others
Other therapies	Immunoglobulin, Low-dose steroid, Renal replacement therapy for renal indication, Renal replacement therapy for cytokine modulation, PMX, Plasmapheresis, VA-ECMO, VV-ECMO, IABP, Procedure for infection, Operation for hemorrhage
Illness severity score on Day 1	APACHE II score, SIRS score, SOFA score lung, SOFA score kidney, SOFA score liver, SOFA score cardiovascular, SOFA score coagulopathy, SOFA score central nervous system
Laboratory data on Day 1	White blood cell count, Platelet count, Hemoglobin, PT ratio, FDP, D-dimer, Lactate
