# Supplementary material for: Comparative Analysis of Three Machine-Learning Techniques and Conventional Techniques for Predicting Sepsis-Induced Coagulopathy Progression
Source: J Clin Med. 2020 Jul 4;9(7):2113. doi: 10.3390/jcm9072113 (PMC7408668; doi:10.3390/jcm9072113)
Supplement: Supplementary file 1 [file jcm-09-02113-s001.zip › TableS2.pdf]

Table S2. Variables included in the analysis, their estimated values, and *p*-value in multiple linear regression analysis with complete data

	**Estimated values**	**SE**	***p*-value**
Pre-existing coagulopathy-related history: Cirrhosis	0.69	0.29	0.019
Bleeding event: Transfusion for bleeding	−0.30	0.18	0.102
Bleeding event: Intracranial hemorrhage	1.52	0.88	0.084
Anticoagulant therapy, unrelated to sepsis-induced coagulopathy: Antithrombin	−0.28	0.12	0.023
Anticoagulant therapy, unrelated to sepsis-induced coagulopathy: Nafamostat	0.61	0.14	< 0.001
Anticoagulant therapy, unrelated to sepsis-induced coagulopathy: Others	−1.57	0.76	0.039
PMX	0.49	0.15	0.001
SOFA score, coagulopathy	−0.31	0.05	< 0.001
SOFA score, central nervous system	0.13	0.04	0.003
White blood cell count	−0.02	0.01	0.002
PT ratio	−0.24	0.09	0.006
Lactate	0.05	0.02	0.006
Infection site: Bone/soft tissue	0.39	0.18	0.025
Infection site: Urinary tract	−0.44	0.18	0.016
Infection site: Lung	0.32	0.15	0.037
Infection site: Others	−0.96	0.38	0.011
Causal pathogen: Gram-positive coccus	−0.30	0.14	0.030

Causal pathogen: Fungus	−0.68	0.36	0.060
Admission route: Medical ward	−0.22	0.13	0.077

SE, standard error; PMX, polymyxin B hemoperfusion; SOFA, Sequential Organ Failure Assessment; PT ratio, prothrombin:time ratio.
