# Supplementary material for: Comparative Analysis of Three Machine-Learning Techniques and Conventional Techniques for Predicting Sepsis-Induced Coagulopathy Progression
Source: J Clin Med. 2020 Jul 4;9(7):2113. doi: 10.3390/jcm9072113 (PMC7408668; doi:10.3390/jcm9072113)
Supplement: Supplementary file 1 [file jcm-09-02113-s001.zip › TableS3.pdf]

Table S3. Variables and their odds ratios in multiple logistic regression analysis with complete data.

	**OR**	**Lower CL**	**Upper CL**	***p*-value**
Age	0.99	0.98	1	0.06
Anticoagulant therapy for sepsis-induced coagulopathy: Antithrombin	0.7	0.47	1.03	0.073
Anticoagulant therapy, unrelated to sepsis-induced coagulopathy: Nafamostat	1.87	1.11	3.15	0.018
Other therapies: Renal replacement therapy for renal indication	1.58	0.96	2.6	0.071
PMX	2.59	1.64	4.07	< 0.001
SOFA score, coagulopathy	0.57	0.46	0.71	< 0.001
SOFA score, central nervous system	1.28	1.12	1.46	< 0.001
White blood cell count	0.98	0.96	1	0.013
Platelet count	1	0.99	1	0.031
PT ratio	0.61	0.43	0.86	0.005
FDP	1	1	1	0.082
Lactate	1.05	1	1.1	0.076
Infection site: Bone/soft tissue	1.99	1.18	3.36	0.01
Infection site: Lung	1.49	0.95	2.34	0.08
Infection site: Others	0.13	0.02	1.06	0.057
Blood culture: Negative	1.34	0.93	1.94	0.12

Causal pathogen: Gram-positive coccus	0.64	0.41	1	0.051
Admission route: Medical ward	0.62	0.41	0.92	0.017

OR, odds ratio; CL, confidence level; PMX, polymyxin B hemoperfusion; SOFA, Sequential Organ Failure Assessment; PT ratio, prothrombin:time ratio; FDP, fibrin/fibrinogen-degradation product
