# Supplementary material for: Comparative Analysis of Three Machine-Learning Techniques and Conventional Techniques for Predicting Sepsis-Induced Coagulopathy Progression
Source: J Clin Med. 2020 Jul 4;9(7):2113. doi: 10.3390/jcm9072113 (PMC7408668; doi:10.3390/jcm9072113)
Supplement: Supplementary file 1 [file jcm-09-02113-s001.zip › TableS4.pdf]

Table S4. Included variables, their estimated value, and *p*-value in multiple linear regression analysis with imputation.

	**Estimated value**	**SE**	***p*-value**
Pre-existing comorbidities: Liver insufficiency	−0.64	0.44	0.143
Pre-existing coagulopathy-related history: Cirrhosis	1.13	0.43	0.009
Pre-existing coagulopathy-related history: Leukemia	0.71	0.29	0.013
Pre-existing coagulopathy-related history: Anti-cancer medication	0.42	0.25	0.091
Pre-existing coagulopathy-related history: Warfarin use	0.55	0.28	0.047
Other therapies: Procedure for infection	−0.15	0.10	0.161
Bleeding event: Intracranial hemorrhage	1.49	0.93	0.108
Other therapies: Operation for hemorrhage	−0.86	0.44	0.048
Anticoagulant therapy for sepsis-induced coagulopathy: Antithrombin	−0.19	0.11	0.077
Anticoagulant therapy for sepsis-induced coagulopathy: Protease inhibitor	0.27	0.14	0.058
Anticoagulant therapy, unrelated to sepsis-induced coagulopathy: Nafamostat	0.30	0.15	0.040
Anticoagulant therapy, unrelated to sepsis-induced coagulopathy: Warfarin	−0.91	0.54	0.094
Anticoagulant therapy, unrelated to sepsis-induced coagulopathy: Antiplatelet	−0.55	0.39	0.161

Anticoagulant therapy, unrelated to sepsis-induced coagulopathy: Others	−1.03	0.53	0.054
Renal replacement therapy for renal indications	0.42	0.14	0.003
PMX	0.45	0.13	0.001
VA-ECMO	1.06	0.72	0.144
VV-ECMO	0.67	0.43	0.121
SOFA score, coagulopathy	−0.41	0.04	0.000
SOFA score, central nervous system	0.11	0.04	0.003
White blood cell count	−0.01	0.00	0.005
PT ratio	−0.26	0.09	0.002
D-dimer	0.00	0.00	0.121
Lactate	0.04	0.01	0.002
Causal pathogen: Gram-positive coccus	−0.19	0.11	0.092
Admission route: Transfer from other hospital	−0.19	0.12	0.117
Admission route: Medical ward	−0.43	0.13	0.001

SE, standard error; PMX, polymyxin B hemoperfusion; VA-ECMO, veno-arterial extracorporeal membranous oxygenation; VV-ECMO, veno-venous extracorporeal membranous oxygenation; SOFA, Sequential Organ Failure Assessment; PT ratio, prothrombin:time ratio
