# Supplementary material for: Comparative Analysis of Three Machine-Learning Techniques and Conventional Techniques for Predicting Sepsis-Induced Coagulopathy Progression
Source: J Clin Med. 2020 Jul 4;9(7):2113. doi: 10.3390/jcm9072113 (PMC7408668; doi:10.3390/jcm9072113)
Supplement: Supplementary file 1 [file jcm-09-02113-s001.zip › TableS5.pdf]

Table S5. Variables and their odds ratios included in multiple logistic regression analysis of imputation.

	**OR**	**Lower CL**	**Upper CL**	***p*-value**
Pre-existing coagulopathy-related history: Cirrhosis	2.42	1.15	5.13	0.021
Pre-existing coagulopathy-related history: Leukemia	3.53	1.63	7.63	0.001
Anticoagulant therapy, unrelated to sepsis-induced coagulopathy: Heparin	0.64	0.41	1	0.051
Other therapy: Renal replacement therapy for renal indications	2.24	1.64	3.06	< 0.001
PMX	1.81	1.3	2.51	< 0.001
SOFA score, coagulopathy	0.63	0.55	0.71	< 0.001
SOFA score, central nervous system	1.19	1.08	1.32	0.001
White blood cell count	0.97	0.96	0.99	< 0.001
PT ratio	0.59	0.44	0.8	< 0.001
Lactate	1.05	1.01	1.09	0.021
Causal pathogen: Virus	0.25	0.05	1.32	0.103
Causal pathogen: Gram-positive coccus	0.69	0.5	0.95	0.024
Admission route: Transfer from other hospital	0.73	0.53	1.02	0.063
Admission route: Medical ward	0.59	0.41	0.83	0.003

OR, odds ratio; CL, confidence level; PMX, polymyxin B hemoperfusion; SOFA, Sequential Organ Failure Assessment; PT ratio, prothrombin: time ratio.
